# Benefits of Self-quantification for Family Caregivers and Future Machine Learning Support

**DOI:** 10.1093/geroni/igaf122.4309

**Published:** 2025-12-31

**Authors:** Tomoko Wakui

**Affiliations:** Tokyo Metropolitan Institute for Geriatrics and Gerontology, Tokyo, Tokyo, Japan

## Abstract

Family caregivers of community-dwelling older adults requiring long-term care often face significant psychological and physical burdens, particularly under the increasingly diverse care arrangements in Japan, where existing support systems are reaching their limits. Self-quantification—systematic daily documentation of caregiving activities and well-being, along with caregivers’ sleep, activity, and health—offers a promising approach to capturing fluctuations in caregiver stress and identifying early risks. The purpose of this study was to comprehensively evaluate the impacts of self-quantification on family caregivers. Specifically, we aimed to elucidate the mechanisms underlying these effects by integrating quantitative findings with qualitative insights from caregivers’ narratives, and examine the potential of applying machine learning to daily caregiving data for predictive risk detection and future digital health support. We developed the Caregiving Visualization Program (Care-VIP), enabling family caregivers to record daily caregiving activities, personal routines, and emotional well-being. An initial online survey of 3,256 caregivers mapped caregiving situations nationwide, followed by a 60-day self-quantification trial with 201 participants. A follow-up survey assessed the program’s perceived benefits and challenges. Program participation was more likely among male caregivers (OR = 1.44), those employed full-time (OR = 1.80), caregivers with longer caregiving duration (OR = 1.17), and those with higher caregiving burden (OR = 1.01). Qualitative feedback indicated that daily documentation fostered reflection, recognition of personal health, and positive behavioral changes, such as the use of respite services and improved health habits. Machine learning analysis of daily sleep/activity data further demonstrated feasibility in detecting patterns predictive of caregiver stress and depressive symptoms.

